# Enhancement of Heterologous Gene Expression in *Flammulina velutipes* Using Polycistronic Vectors Containing a Viral 2A Cleavage Sequence

**DOI:** 10.1371/journal.pone.0059099

**Published:** 2013-03-14

**Authors:** Yu-Ju Lin, Li-Hsin Huang, Ching-Tsan Huang

**Affiliations:** Department of Biochemical Science and Technology, National Taiwan University, Taipei, Taiwan; University of Wisconsin – Madison, United States of America

## Abstract

*Agrobacterium tumefaciens*-mediated transformation for edible mushrooms has been previously established. However, the enhancement of heterologous protein production and the expression of multi-target genes remains a challenge. In this study, heterologous protein expression in the enoki mushroom *Flammulina velutipes* was notably enhanced using 2A peptide-mediated cleavage to co-express multiple copies of single gene. The polycistronic expression vectors were constructed by connecting multi copies of the enhanced green fluorescent protein (*egfp*) gene using 2A peptides derived from porcine teschovirus-1. The P2A peptides properly self-cleaved as shown by the formation of the transformants with antibiotic resistant capacity and exciting green fluorescence levels after introducing the vectors into *F. velutipes* mycelia. The results of western blot analysis, epifluorescent microscopy and EGFP production showed that heterologous protein expression in *F. velutipes* using the polycistronic strategy increased proportionally as the gene copy number increased from one to three copies. In contrast, much lower EGFP levels were detected in the *F. velutipes* transformants harboring four copies of the *egfp* gene due to mRNA instability. The polycistronic strategy using 2A peptide-mediated cleavage developed in this study can not only be used to express single gene in multiple copies, but also to express multiple genes in a single reading frame. It is a promising strategy for the application of mushroom molecular pharming.

## Introduction

The expression of heterologous genes in organisms can be extensively applied in agriculture, industry, bioremediation, and molecular pharming. The production of pharmaceutically and commercially valuable proteins, such as enzymes, vaccines, antibodies, and hormones, by genetically modified plants is known as molecular pharming [1], [2]. In addition to plants, mushrooms are also considered an appropriate host for the production of recombinant proteins. Mushrooms have all the advantages of plant expression systems coupled with unique benefits, including post-translational modification that is more similar to that in animals than plants [3], [4], established scaled-up production under controlled conditions, and a reduced risk of gene contamination. A stable transformation method for mushroom expression systems has been established using *Agrobacterium tumefaciens*-mediated transformation (ATMT) [5], [6]. However, the development of mushroom molecular pharming has been limited due to low transgenic expression levels.

The expression level of recombinant proteins in mushrooms is critical to the success of mushroom molecular pharming. It is the most important factor affecting the cost of cultivation, processing, extraction, purification and waste disposal [7]. Although ATMT has demonstrated the stability of integrating DNA in the mushroom genome, the transgene copy numbers are usually low [8]. Hence, the enhancement of heterologous gene expression in mushrooms is urgently required. Many efforts have been made to enhance the production of heterologous proteins by inserting an intron or an endoplasmic reticulum retention signal peptide [9]–[11] as well as by optimizing codon usage [12], [13]. An alternative approach to enhance gene expression involves increasing gene copy numbers using polycistronic strategies.

Polycistronic strategies, such as fusion proteins, bidirectional promoters, and internal ribosome entry sites (IRESs) [14], can be applied to co-express multiple copies of single gene or multiple genes (polycistrons) via a single transcriptional unit. Additionally, 2A peptides and 2A peptide-like sequences have been utilized in polycistronic strategies [15]. They can be used to mediate the cleavage of polyproteins, which is referred to as 2A peptide-mediated cleavage, to yield equimolar expression levels of separate proteins translated from a polycistronic mRNA [16]–[18]. Previous studies have revealed that 2A peptides can be successfully applied for a broad range of organisms, including yeasts [18], plants [19], [20], mammalian cells [21], [22], and transgenic animals [23].

In this study, we demonstrate that heterologous protein expression in the enoki mushroom *F. velutipes* is notably enhanced using 2A peptide-mediated cleavage to co-express multiple copies of a single gene. The relationship between the copy number of gene insertions and expression levels was also investigated.

## Materials and Methods

### Strains and media

*Flammulina velutipes* BCRC 37086 was purchased from Bioresources Collection and Research Center (Hsinchu, Taiwan) and was grown and maintained on CYM agar or broth (Difco, Detroit, MI) containing 0.2% yeast extract, 0.2% tryptone, 1% maltose and 2% glucose at 25°C. *Escherichia coli* DH5α cells, which were used for DNA manipulation and plasmid conservation, were grown in LB medium (Sigma Chemical Co., St Louis, MO) at 37°C. The *Agrobacterium tumefaciens* strain LBA4404 used to transform *F. velutipes* was kindly provided by Dr. Yee-Yung Charng, Agricultural Biotechnology Research Center, Academia Sinica (Taipei, Taiwan) and grown in LB medium at 30°C.

### Plasmids construction

**Figure 1:**
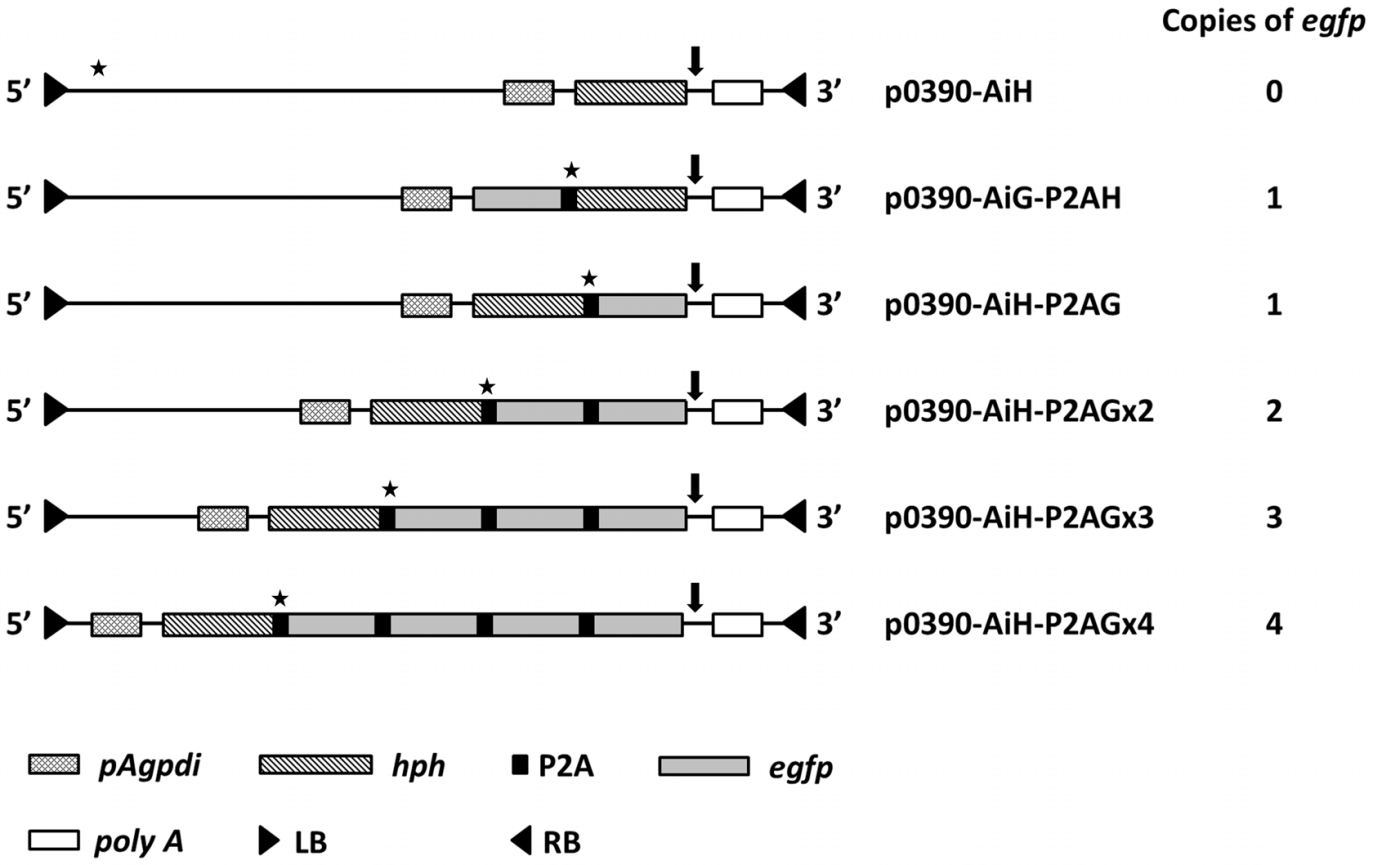
A map of the plasmid constructions. *pAgpdi*: the *gpd* promoter with the *gpd* first intron derived from *A. bisporus*. *egfp*: enhanced green fluorescent protein. *hph*: hygromycin B phosphotransferase gene derived from *E. coli*. P2A: 2A peptides derived from porcine teschovirus-1. *poly A*: nopaline synthase polyA signal. LB: left border. RB: right border. The star indicates the *Bam*HI restriction enzyme site and the arrowhead indicates the *Bgl*II restriction enzyme site used for southern blot analysis.

The backbone plasmid p0390-AiH harboring the *E. coli* hygromycin B phosphotransferase (*hph*) gene under the control of the *gpd* promoter of *Agaricus bisporus* was constructed by inserting the p*gpd-hyh* cassette [24] into the pCAMBIA0390 vector (http://www.cambia.org.au). The codon usage of P2A peptides derived from porcine teschovirus-1 [25] was modified according to the codon bias of *F. velutipes*. The enhanced green fluorescent protein (*egfp*) gene was used to assess gene expression. All PCR products were digested using the appropriate restriction enzymes and ligated into the p0390-AiH vector to generate the constructs p0390-AiH-P2AG, p0390-AiH-P2AGx2, p0390-AiH-P2AGx3, p0390-AiH-P2AGx4. To compare the upstream and downstream gene expression of P2A, the p0390-AiG-P2AH vector was also constructed. The plasmids were introduced into *A. tumefaciens* via electroporation using BTX ECM 63 (BTX, San Diego, CA). A map of the plasmid constructs is showed in Figure 1, and the primers used in this study are listed in Table 1.

**Table 1:** Primers used in this study.

Name	Sequence	Usage
Api-f	5′-CCAAGCTTTTAAGAGGTCCGCAAGTAG-3′	*Agaricus bisporus gpd* promoter with
Api-r	5′-CGCTGCAGCTACAAGTCGACATCAGTG-3′	the first intron cloning
P2A-f	5′-CGGGATCCGCCTTCCAAGGCCCCGGCGCCACCAACTTCTCTCTCCTC-3′	2A peptides from porcine
P2A-r	5′-GGGCCCGGGGTTCTCCTCGACGTCGCCGGCCTGCTTGAGGAGAGAGAA-3′	teschovirus-1 cloning
eGFP-f	5′-GCTGCAGGTGAGCAAGGGCGAGGAGC-3′	*egfp* cloning
eGFP-r	5′-GGGTCACCTTAAGATCTCTTGTACAGCTCGTCCAT-3′	
eGFP-362f	5′-TGAACCGCATCGAGCTGAAGGG-3′	Real-time PCR analysis
eGFP-468r	5′-ACCTTGATGCCGTTCTTCTGCTTG-3′	
eGFP-QF	5′-GATGTTGTGGCGGATCTT-3′	Quantitative RT-PCR
eGFP-QR	5′-TTGTACTCCAGCTTGTGC-3′	
Hyg-f	5′-CGCTGCAGAAAAAGCCTGAACTCACC-3′	*hph* cloning
Hyg-r	5′-CGGGATCCCTATTTCTTTGCCCTCGGAC-3′	

### Transformation procedure

ATMT was performed as described by Chen *et al*. [6] with minor modification. The *A. tumefaciens* strains harboring different constructs were pre-induced in 200 μM acetosyringone (AS) with *F. velutipes* modified mycelia pellet (MMP) and incubated for 6 h at 30°C. After incubation, the resulting mixture of *F. velutipes* and *A. tumefaciens* was co-cultivated at 23°C for 3–6 days. After co-cultivation, the treated MMPs were washed with sterile water five times to remove *Agrobacterium* and then transferred to selection agar plates containing 30 μg/mL hygromycin and 200 μM cefotaxime (MDBio, Taipei, Taiwan) at 25°C. The plates were incubated at 23°C for 2–3 weeks until hygromycin B-resistant mycelia of *F. velutipes* appeared.

### Real-time PCR

The modified genomic DNA extraction procedure has been described by Doyle *et al*. [26]. The KAPA^TM^ SYBR FAST Universal qPCR kit (Kapa Biosystems, Boston, MA) and the MiniOpticon real-time PCR system (Bio-Rad, Hercules, USA) were used under the following cycling conditions: a denaturing stage of 95°C for 1 min followed by 95°C for 5 sec and 60°C for 30 sec for the cycling stage of 40 cycles. The *egfp* primers are listed in Table 1. The target and reference genes were amplified on the same plate in triplicate. The amplification product was 110 bp in length for the *egfp* gene, and the Ct values were calculated using CFX Manager 1.6 software (Bio-Rad, Hercules, CA).

### Southern blot

Southern blotting was performed based on the method described by Kuo *et al*. [27]. Briefly, 10 μg of genomic DNA derived from each transformant was digested with the restriction enzymes *Bgl*II and *Bam*HI, separated on a 0.8% agarose gel and vacuum transferred to an Immobilon-Ny+ transfer membrane (Amersham, Hong Kong). The *egfp* probe was generated using the EGFP-F and EGFP-R primers. Subsequent probe labeling, hybridization, and signal detection were conducted using a digoxigenin (DIG)-probe synthesis and detection kit (Roche, Mannheim, Germany) according to the manufacturer's instructions.

### RNA extraction and cDNA synthesis

For RNA analysis, 8-week-old mycelia of wild type and transformant *F. velutipes* were collected and subsequently ground in liquid nitrogen. Total RNA derived from the cells was extracted using TRIzol reagent (Invitrogen, Carlsbad, CA) in accordance with the manufacturer's procedure. To avoid RNA degradation and DNA contamination, the total RNA was treated with RNase OUT and DNase I (Invitrogen). Reverse transcription was performed using SuperScript^TM^ II Reverse transcriptase (Invitrogen) with oligo-dT18 (Genemark, Taipei, Taiwan), and the products were stored at −20°C for subsequent quantitative RT-PCR assays.

### Quantitative RT-PCR

The standard supercoiled plasmid DNA was prepared as previously described [28]. The dsDNA concentrations were estimated using a NanoDrop 1000 Spectrophotometer (Thermo Scientific, Asheville, NC). The reference plasmid was prepared as calibration curves by generating ten-fold serial dilutions. After performing the reverse transcription reaction, cDNA was used for quantitative PCR in which the SYBR green system (Bio-Rad) and the KAPA^TM^ SYBR FAST Universal qPCR kit (Kapa Biosystems, Boston, MA) were used for the reactions. The sense and antisense primers are listed in Table 1. The PCR conditions applied were as follows: 95°C for 30 sec for the denaturing stage followed by 95°C for 5 sec and 60°C for 30 sec for the cycling stage of 40 cycles, which was followed by melting curve analysis.

### Western blot

For western blot analysis, *F. velutipes* transformants and wild type strain were grown in CYM broth for a month. Mycelia were collected and subsequently ground in liquid nitrogen with a mortar and pestle. A total of 30 mg of mycelial powder was mixed with 600 μL protein extraction buffer (50 mM sodium phosphate, 300 mM NaCl, 1 mM PMSF, and 0.1% Triton X-100, pH 7.4) on ice for 1 h. After centrifuging the mixture twice at 13,000 *g* for 30 min, the supernatant was collected, which represented the total soluble protein (TSP). The protein samples were separated via 12% sodium dodecyl sulfate polyacrylamide gel electrophoresis (SDS-PAGE). The protein samples were transferred via semi-dry blotting (Genmedika, Taipei, Taiwan) to a PVDF membrane (PerkinElmer, Boston, MA). Protein detection was performed using a monoclonal anti-GFP living-color peptide antibody (BD Bioscience) and colorimetric detection of the target pattern was performed with the NBT/BCIP reaction (PerkinElmer) as described by the manufacturers.

### EGFP quantification

Mycelia were collected and subsequently ground in liquid nitrogen with a mortar and pestle. Thirty mg mycelial powder was mixed with 600 μL protein extraction buffer (50 mM sodium phosphate, 300 mM NaCl, 1 mM PMSF, and 0.1% Triton X-100, pH 7.4) on ice for a period of 1 h. After centrifuging at 13,000 *g* for 30 min, the supernatant was collected as the TSP. A sandwich ELISA for EGFP detection was performed as described by Kuo *et al*. [27]. The samples were incubated for 1 h on ELISA plates (PerkinElmer) coated with monoclonal EGFP antibody (ab1218, Abcam, Cambridge, UK). Each sample assay was repeated in triplicate for each plate. Rabbit anti-GFP polyclonal antibody (ab6556, Abcam) was added to each well at a 1:6,000 dilution and incubated for 1 h at 4°C. Goat polyclonal antibodies against rabbit IgG conjugated to HRP enzyme (PerkinElmer) were added to each well at a 1:5,000 dilution and incubated for 1 h at 4°C. Next, 100 μL of TMB-HRP microwell substrate (BioFX, Owings Mills, MD) was added to each well. After 5 min, the absorbance at 650 nm in each well was measured using a 96-well plate reader (VERSAmax, Sunnyvale, CA). Protein concentrations were determined using the BCA^TM^ Protein Assay Kit (Pierce, Rockford, IL). The commercially available EGFP protein (BioVision, Milpitas, CA) was used as a standard.

### Fluorescent microscopy

To analyze *egfp* expression, the mycelia of *F. velutipes* transformants were transferred to glass slides. After growth for 5 days, the samples were analyzed using a fluorescence microscope (E600, Nikon, Kanagawa, Japan) fitted with a Nikon B-2A filter (450–490 nm excitation filter; 505 nm dichroic filter; and 520 nm barrier filter).

## Results

### Transformation procedure

*F. velutipes* MMPs were co-cultivated with *A. tumefaciens* containing p0390-AiH, p0390-AiG-P2AH, p0390-AiH-P2AG, p0390-AiH-P2AGx2, p0390-AiH-P2AGx3, p0390-AiH-P2AGx4 in inductive media for 5–7 days and transferred to selection agar plates. The hygromycin B-resistant mycelia appeared approximately 2–4 weeks after transfer to selection agar plates, while MMPs co-cultivated with. *A. tumefaciens* carrying the negative control pCAMBIA 0390 failed to grow. The sub-cultured mycelia stably grew on selectable agar plates. The results indicated that P2A peptide-mediated cleavage was applicable in *F. velutipes* as shown by the formation of transformants with both hygromycin B resistance and green fluorescence.

### *F. velutipes* transformant nucleotide analysis

**Figure 2:**
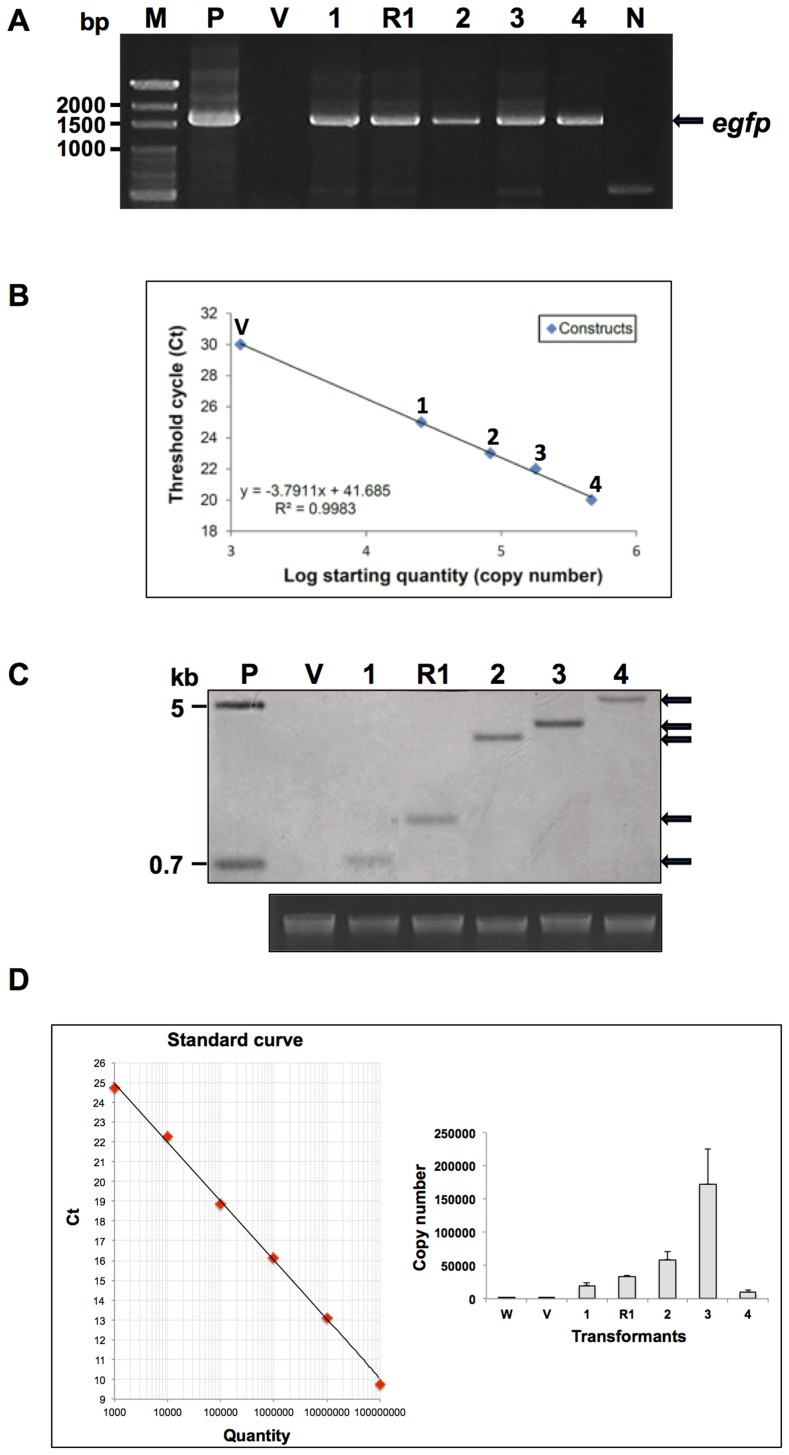
Analysis of nucleotides isolated from putative transformants. (A) PCR. (B) Real-time PCR. (C) Southern blot. (D) Quantitative RT-PCR. Lane P: positive control using plasmid DNA; lane W: *F. velutipes* wild type; lane V: *F. velutipes* AiH transformant vector control; lane 1: *F. velutipes* AiH-P2AG transformants; lane R1: *F. velutipes* AiG-P2AH transformants; lane 2: *F. velutipes* AiH-P2AGx2 transformants; lane 3: *F. velutipes* AiH-P2AGx3 transformants; lane 4: *F. velutipes* AiH-P2AGx4 transformants; lane N: negative control; and lane M: marker. The quantitative RT-PCR experiments were repeated three times. The error bars indicate the standard error.

*F. velutipes* transformants grown on selective agar plates were subsequently screened via PCR analysis. The amplified DNA products, which contained the *A. bisporus gpd* promoter with the first intron and the *egfp* gene, were approximately 1500 bp in length (Figure 2A). The copy number of the *egfp* gene for each construct was assessed via real-time PCR. The results showed that no PCR products were amplified with the p0390-AiH and pCAMBIA 0390 vectors, while the PCR product quantity for the p0390-AiH-P2AG, p0390-AiH-P2AGx2, p0390-AiH-P2AGx3, and p0390-AiH-P2AGx4 constructs was proportional to the copy numbers (Figure 2B). Southern blot analysis of each polycistronic transformant, as illustrated in Figure 2C, showed that *egfp* integrated into the genome and that the fragment sizes coincided with the copy numbers.

Quantitative RT-PCR analysis of the *egfp* transcripts was performed for the p0390-AiH, p0390-AiH-P2AG, p0390-AiG-P2AH, p0390-AiH-P2AGx2, p0390-AiH-P2AGx3, p0390- AiH-P2AGx4 constructs on 8-week-old *F. velutipes* transformants and the wild type controls. The analysis revealed that under the same cultivation conditions, the p0390-AiH, p0390-AiH-P2AG, p0390-AiH-P2AGx2, p0390-AiH-P2AGx3 constructs showed a tendency of increased expression levels (Figure 2D), while the p0390-AiH-P2AGx4 levels were relatively low. Thus, a dramatic difference in gene expression was observed with *egfp* constructs of up to four copies. Gene expression also assessed in the constructs with *egfp* upstream and downstream of P2A. It was found that *egfp* upstream of P2A caused an increase in gene expression. This reveals the importance of gene position that is directly driven by the promoter.

### Western blot analysis and fluorescent microscopic observations

**Figure 3:**
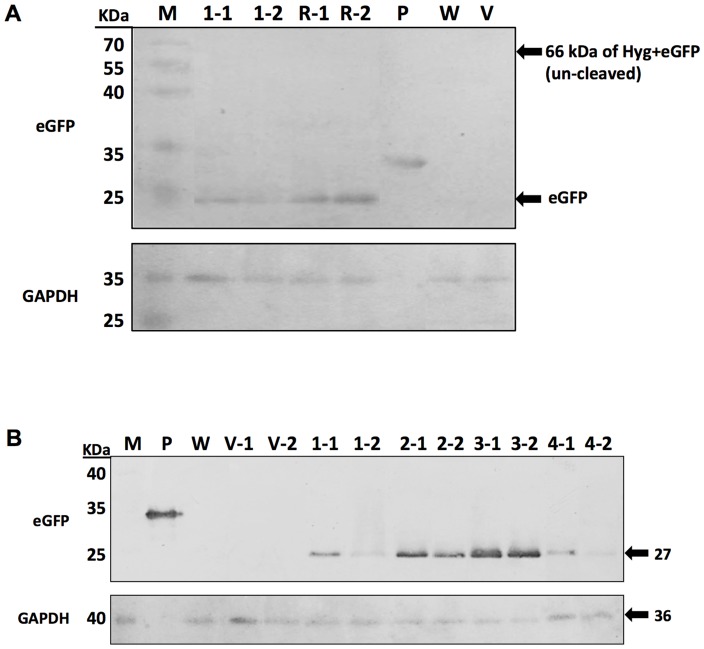
Expression of EGFP in *F. velutipes* transformants. (A) Western blot analysis of EGFP protein expression with the various gene positions in the *F. velutipes* transformants AiH-P2AG and AiG-P2AH. (B) Western blot analysis of EGFP protein expression with the various copy numbers in the indicated *F. velutipes* transformants. Lane P: positive control, a commercial EGFP containing tags at both the N and C terminus (293 amino acids, 32.7 kDa), the EGFP expressed in transformants is 266 amino acids, 27 kDa; lane W: wild type; lane V-1, V-2: *F. velutipes* AiH transformant vector control; lane 1–1, 1–2: *F. velutipes* AiH-P2AG transformants; lane 2–1, 2–4: *F. velutipes* AiH-P2AGx2 transformant; lane 3–1, 3–2: *F. velutipes* AiH-P2AGx3 transformants; lane 4–1, 4–2: *F. velutipes* AiH-P2AGx4 transformants; and lane M: marker.

**Figure 4:**
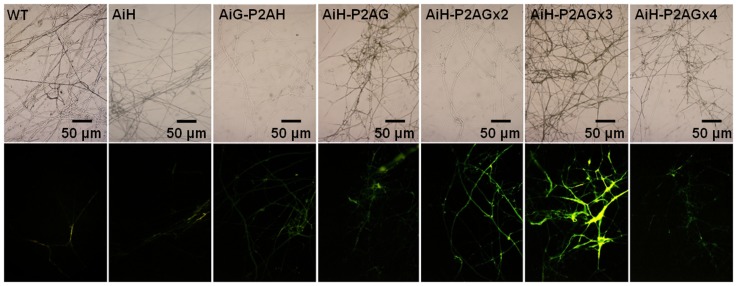
Microphotographs of representative transformant mycelia using bright field (top) and epifluorescent (bottom) microscopy under the same exposure condition (0.25 sec).

The western blot analysis results of *F. velutipes* transformants with the various *egfp* gene positions are shown in Figure 3A. The results are consistent with the results obtained from Quantitative RT-PCR analysis. The results indicate that EGFP expression increases when *egfp* is located upstream of P2A. Additionally, no un-cleaved form was observed on the membrane. The various copy numbers for each *F. velutipes* transformant were confirmed via western blot analysis (Figure 3B). Signal intensity increased proportionally with the increase in copy numbers (p0390-AiH-P2AG<p0390-AiH-P2AGx2<p0390-AiH-P2AGx3), while only weak signals were found for the transformants of p0390-AiH-P2AGx4. The representative epifluorescent microphotographs of transformants harboring different constructions are shown in Figure 4. The mycelia of *F. velutipes* for the wild type and transformants carrying p0390-AiH exhibited weak yellow autofluorescence. In contrast, green fluorescence was observed with the mycelia of *F. velutipes* transformants of p0390-AiH-P2AG and p0390-AiG-P2AH. With increasing copy numbers of *egfp*, the p0390-AiH-P2AGx3 transformants emitted the most intense green fluorescence followed by the p0390-AiH-P2AGx2 transformants. Only weak green fluorescence was observed in the p0390-AiH-P2AGx4 transformants. The results of the fluorescent microscopic observations are consistent with the western blot results.

### EGFP quantification via ELISA

**Figure 5:**
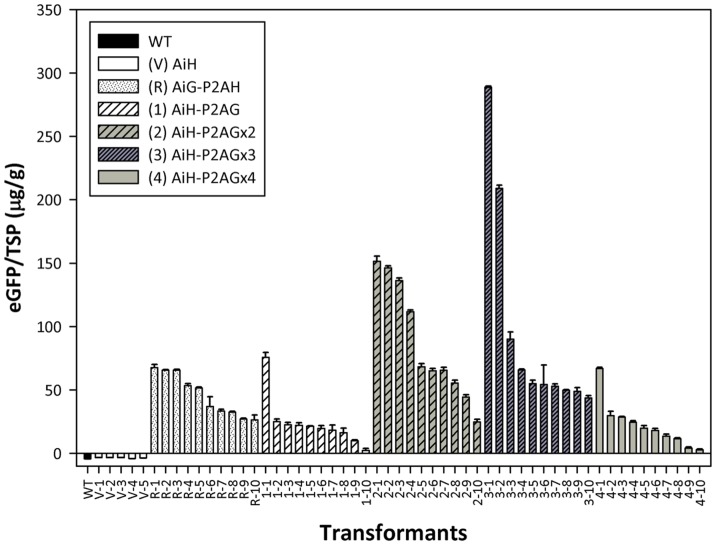
EGFP production of mycelia for wild type and transformant *F. velutipes*. TSP: total soluble protein. The EGFP assays were repeated three times. The error bars indicate the standard deviation.

EGFP expression for each polycistronic transformant was further evaluated via ELISA. Transformants confirmed via fluorescent microscopic observation were selected for EGFP assays. Mycelia of ten randomly selected transformants for various copies of the *egfp* constructs were washed free of medium, frozen and ground into fine powder, which was followed by protein extraction. Figure 5 shows the EGFP production of the transformants for each construct. Although heterologous protein production varied among the transformants, EGFP production levels of the p0390-AiG-P2AH transformants were higher than those observed with p0390-AiH-P2AG. Additionally, EGFP production levels in general increased as the copy number increased in the transformants (p0390-AiH-P2AG<p0390-AiH-P2AGx2<p0390-AiH-P2AGx3), while the production of EGFP in the p0390-AiH-P2AGx4 transformants was similar to that of p0390-AiH-P2AG. Both the quantitative and qualitative analyses indicate that the P2A peptide-mediated cleavage system enhances heterologous protein expression.

## Discussion

In many organisms, 2A peptides facilitate the long-term and stable co-expression of multiple genes [29]. In this report, we successfully established a feasible polycistronic expression system in *F. velutipes* using P2A peptides. *egfp* expression was enhanced with the polycistronic strategy, and protein production was proportional to the copy numbers up to three copies. However, notably reduced EGFP levels were detected in the *F. velutipes* transformants harboring four copies of the *egfp* gene. This phenomenon might be attributed to the reasons outlined below.

Transcriptional and translational output might be proportional to DNA copy number under certain circumstances [30]. The polycistron uses a single promoter to drive the same mRNA transcript with two or more coding areas (cistrons). The promoter, mRNA stability and cistron numbers are the critical factors of gene expression. Promoter strength played an important role in target mRNA production. In this study, the 276-bp long *gpd* promoter derived from *A. bisporus* was used to drive the expression of the heterologous gene. It is strong enough to drive to transcription of the 1011-bp long homologous GPD gene. Therefore, the *gpd* promoter derived from *A. bisporus* may be able to drive the expression of up to three copies of the *egfp* gene. In contrast, the *gpd* promoter from *A. bisporus* may be unable to drive the expression of the 3220-bp long four tandem repeats of *egfp* with P2A plus the first intron. This observation is consistent with other studies, which have shown that heterologous expression and gene transformation efficiency are affected by different basidiomycete promoter fragments and sizes [31], [32].

The stability or half-life of mRNA also played an important role in controlling gene expression. Previous studies have shown a significant negative correlation between mRNA length and stability in some prokaryotes and eukaryotes. The longer the mRNA fragment, the more likely endonucleolytic attacks by RNA endonuclease and/or mechanical damage [33], [34]. The four copies of P2A-*egfp* plus the first intron might be too long to maintain mRNA stability. The relationship between mRNA length and heterologous gene expression still requires further investigation.

The effect of a target gene located at upstream or downstream of the 2A peptides was also investigated in this study. In general, the EGFP expression levels detected in the transformants harboring p0390-AiG-P2AH were higher than in those harboring p0390-AiH-P2AG. This effect was because EGFP upstream of P2A was directly driven by the promoter, while EGFP downstream of P2A was derived via the cleavage mechanism. The excess of ‘cleavage’ product N-terminal of P2A over the product C-terminal of P2A might also be explained by the efficiency of the ‘pseudo-termination’ event and ‘re-initiation’ event [25], [35].

In conclusion, a polycistronic strategy using P2A peptides was developed to enhance heterologous gene expression in *F. velutipes*. This strategy can not only be used to express a single gene in multiple copies, but can also be used to express multiple genes in a single reading frame. It is a promising strategy for the application of mushroom molecular pharming.
